# Non-Destructive Measurement of the Pumpkin Rootstock Root Phenotype Using AZURE KINECT

**DOI:** 10.3390/plants11091144

**Published:** 2022-04-23

**Authors:** Moran Zhang, Shengyong Xu, Yuan Huang, Zhilong Bie, Michitaka Notaguchi, Jingyi Zhou, Xin Wan, Yuchen Wang, Wanjing Dong

**Affiliations:** 1 College of Engineering, Huazhong Agricultural University, Wuhan 430070, China; moran@webmail.hzau.edu.cn (M.Z.); xsy@mail.hzau.edu.cn (S.X.); wanxin@webmail.hzau.edu.cn (X.W.); 2 Key Laboratory of Agricultural Equipment for the Middle and Lower Reaches of the Yangtze River, Ministry of Agriculture, Wuhan 430070, China; 3 College of Horticulture and Forestry Sciences, Huazhong Agricultural University, Wuhan 430070, China; huangyuan@mail.hzau.edu.cn (Y.H.); biezl@mail.hzau.edu.cn (Z.B.); notaguchi.michitaka.c4@f.mail.nagoya-u.ac.jp (M.N.); zhoujingyi@webmail.hzau.edu.cn (J.Z.); wyc2002@webmail.hzau.edu.cn (Y.W.); 4 Key Laboratory of Horticultural Plant Biology, Ministry of Education, Wuhan 430070, China; 5 Bioscience and Biotechnology Center, Nagoya University, Nagoya 464-8601, Japan

**Keywords:** grafting seedlings, roots, phenotype, pumpkin, RGBD

## Abstract

Rootstock grafting is an important method to improve the yield and quality of seedlings. Pumpkin is the rootstock of watermelon, melon, and cucumber, and the root phenotype of rootstock is an important reference for breeding. At present, the root phenotype is mainly measured by scanners, with which it is difficult to achieve non-destructive and in situ measurements. In this work, we propose a method for non-destructive measurement of the root phenotype on the surface layer of the root ball of pumpkin rootstock plug seedlings and an accurate estimation of the surface area, length, and volume of total root using an AZURE KINECT sensor. Firstly, the KINECT is used to capture four-view color and depth images of the root surface, and then multi-view images are spliced to obtain a complete image of the root surface. After preprocessing of the images, we extract the roots from the root ball. For root phenotype measurements, the surface areas of the surface roots and root ball are calculated, followed by calculating root encapsulation. Next, the non-overlapping roots in the surface root image are extracted, and the ratio of the surface area to the skeleton length is used as the average diameter of total root. Based on the high correlation between the surface area of surface root and the surface area of total root, a linear fitting model is established to estimate the surface area, length, and volume of total root. The experiment ultimately showed that the measurement error for the average diameter of total root is less than 30 μm, and consistency with the scanner is higher than 93.3%. The accuracy of the surface area of total root estimation was found to be more than 88.1%, and the accuracy of the root length of total root estimation was observed to be greater than 87.2%. The method proposed in this paper offers similar accuracy to a scanner, which meets the needs of non-destructive root phenotype research. This method is expected to replace root scanners for high-throughput phenotypic measurements and provides a new avenue for root phenotype measurements of pumpkin rootstocks. This technology will provide key basic data for evaluating the root growth of pumpkin rootstocks.

## 1. Introduction

Grafting is a widely used technique in the production of fruit-bearing vegetables [1]. Pumpkin has the characteristics of stress resistance and vigorous growth and is widely used as a rootstock for watermelon, melon, and cucumber in production. Pumpkin rootstock grafting can improve the nutrient absorption and water-use efficiency of watermelon and other crops [2,3]. The root phenotype is closely related to water and nutrient uptake and the stress resistance of rootstocks. Root length, root number, surface area of total root, etc., can be used to characterize the intrinsic absorption capacity for nutrients and water [4]. Further root phenotypes such as the average diameter of total root, surface area, and root length of total root can be used to evaluate the root morphology of rootstocks [5]. Therefore, non-destructive determination of the root phenotype of rootstocks is very important for cultivar selection.

Studies must identify important root parameters through modeling to simplify the characterization of the root system [6]. At present, measuring the root phenotype of rootstock mainly relies on manual tools and instruments, such as a measuring ruler, the three-dimensional root coordinate container method, the plant root chamber isotope tracer method, the fine root chamber sampling measurement, and root scanners [7]. Artificial measurements can only offer destructive sampling detection, not the continuous observation of plants. Moreover, such measurements are time-consuming. Non-destructive measurement technology for roots has thus become a hot topic in the phenotypic field. X-ray [8], MRI [9], thermal pulse [10], micro-root tube [11], ground penetrating radar [12], root box [13], and other methods have been studied and applied. These non-destructive measurement methods have created a new direction in root research. Compared to these active detection methods, visible light imaging technology is often used to build low-cost and high-throughput non-destructive testing systems. For example, Wang et al. proposed an image-based high-throughput system that integrates simple and reliable root image acquisition hardware and an automatic root image processing algorithm [14]. Hui et al. developed an image-based semi-automatic root phenotype method for field crops; this method can be applied to the three-dimensional reconstruction of the root structure in field growth to improve the input of data-driven models seeking to simulate root growth, solute transport, and water absorption [15]. Yuko et al. developed a new automatic analysis method for digital photos; this method is suitable for identifying adventitious roots as a feature of the root structure in root canals [16]. Since visible light cannot penetrate the measured object, this method can only be used for the non-destructive measurement of surface roots that emerged on the surface of the soil compounds. However, the phenotypic parameters of such surface roots on the soil compounds are closely related to the complete roots included in the soil compounds, which is of great significance for understanding the structure and function of plant roots. For example, Wu et al. found, through non-destructive testing of the surface root traits of rice, that the ratio of the surface root to aboveground area had a similar normal distribution, which played a certain role in crop yield estimation and breeding [17].

The root phenotype of the rootstock is important, but there is currently no relevant non-destructive testing technology available. Instead, a root scanner is widely used. This destructive and cumbersome measurement method greatly limits the advancement of root related research. To take into account the comprehensiveness, accuracy, and high throughput of the measurement, this paper proposes a non-destructive measurement and complete root phenotype estimation method for the surface root phenotype of plug seedlings using an RGBD camera. We also carried out related research on widely used pumpkin rootstocks. Firstly, an Azure Kinect sensor, a sensor for RGBD provided (generated) by Microsoft was used to capture color and depth images of the four perspectives of pumpkin rootstock roots based on a two-dimensional code pattern. Then, according to two-dimensional code feature detection and matching, RGBD images of the four perspectives are spliced into a whole image containing the complete surface root. After that, the root nodules are segmented from the background using the edge segmentation algorithm based on the maximum connected domain, and an image segmentation algorithm based on homomorphic filtering and the FRANGI [18] method is designed to separate the surface root from the root ball. Then, the root length, surface area, average diameter, and encapsulation (root area to soil area ratio) of the surface root are calculated, and the surface area, length, and volume of the total root are estimated. The experimental results showed that this method provides good measurement accuracy for many varieties of pumpkin rootstocks and represents a useful example of low-cost and high-throughput non-destructive measurement technology for seedling roots.

## 2. Materials and Methods

### 2.1. Experimental Location, Equipment, and Design

**Figure 1:**
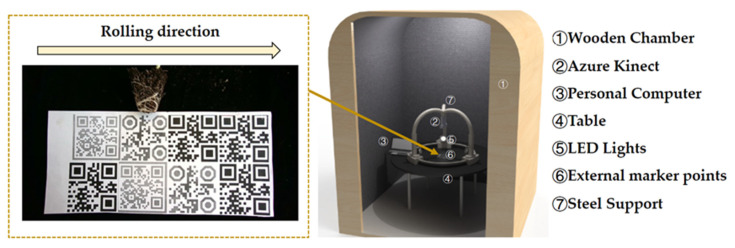
Image acquisition schematic.

The experiment was conducted in a plant growth room in 2021 at the National Center of Vegetable Improvement at Huazhong Agricultural University, Central China (30°270′ N, 114°200′ E and an altitude of 22 m above sea level). In this experiment, we used the random sampling of six varieties of pumpkin seedlings, with 15–20 randomly selected seedings from each cultivar taken as the experimental object. Image acquisition was performed 14 days after the emergence of seedlings. The image acquisition and algorithm development platform used in this study mainly included an Azure Kinect sensor, a black curtain (posted 2D code), three constant light sources, a darkroom, and a general-purpose computer [Core i5-9300H (Intel Corporation, Santa Clara, America) / 8G (SAMSUNG, Seoul, Korea) / 1T (SAMSUNG, Seoul, Korea) / GTX1650 (NVIDIA, Santa Clara, America) / Windows 10 Operating System (Microsoft, Seattle, America)]. The programming environments were Python/MATLAB2019a and the OpenCV4.0/computer vision toolbox, with GPU parallel computing used for acceleration. The image acquisition method is shown in Figure 1. The seedlings were placed in the center of the darkroom in front of the curtain. Manual operation of the computer program was used to control the Azure Kinect when shooting the image. Each shot was obtained by manually rotating the plant sample 90°, and a total of four groups of RGBD images were taken (color image resolution of 1280 × 720, depth image resolution of 1280 × 720). After the root images were taken, the root was washed with water, scanned with an Epson Expression 12000XL scanner (Epson Corporation, Nagano, Japan), and analyzed with the WinRHIZO Pro software. A series of phenotypic parameters were then measured, including the root average diameter, surface area, and root length of total root. The data obtained by the root scanner were subsequently used as the reference values for algorithm verification.

### 2.2. Growth Conditions and Plant Materials

During cultivation, the day and night temperatures were 28 and 18 °C, respectively; the photosynthetic photon flux density was 170 μmol·m^−2^·s^−1^, with a 14/10 h photoperiod; and the daytime relative humidity was 65–85%. The sum of irrigated water for each plug tray was 3.3 L during vegetation growth. Plants were fertilized with water-soluble fertilizer (Product number: 20-10-20 + TE, 1000 times liquid, Hubei Greencare Agriculture Co., Ltd., Wuhan, China). Pumpkin varieties used in this study included cv. ‘Qingyan Rootstock No.1’ (Qingdao Academy of Agricultural Sciences, Qingdao, China), ‘Cucumber Rootstock RC1901’ (Qingdao Golden Ma Ma Agricultural Science and Technology Co., Ltd., Qingdao, China), ‘Cucumber Rootstock RC1902’ (Qingdao Golden Ma Ma Agricultural Science and Technology Co., Ltd., Qingdao, China), ‘Cucumber Rootstock RC1903’ (Qingdao Golden Ma Ma Agricultural Science and Technology Co., Ltd., Qingdao, China), ‘Cucumber Rootstock RC1904’ (Qingdao Golden Ma Ma Agricultural Science and Technology Co., Ltd., Qingdao, China), and ‘Cucumber Rootstock RC1905’ (Qingdao Golden Ma Ma Agricultural Science and Technology Co., Ltd., Qingdao, China). Seventy-two seedlings were cultivated for each variety. From among all prepared seedlings, 50 seedlings were randomly chosen and analyzed.

### 2.3. Multi-View Color-Image and Depth-Image Mosaic Method for the Surface Root System of Root Ball of Pumpkin Rootstock Based on External Feature Points

**Figure 2:**
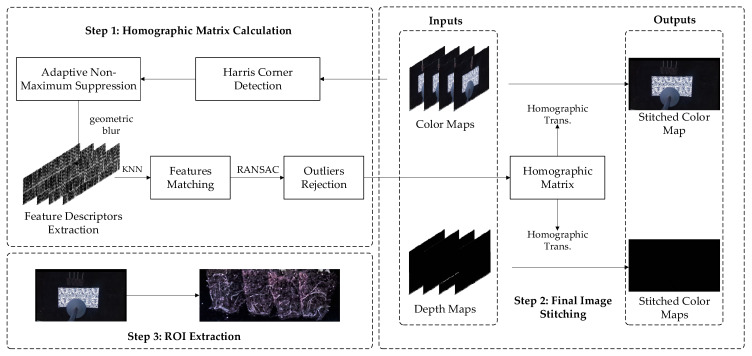
Multi-view RGBD image stitching process for the surface root system. Step 1: Homographic Matrix Calculation. Step 2: Final Image Stitching. Step 3: ROI Extraction.

Stitching four surface root images from four angles into one image is the premise of complete and accurate root measurement. The feature-based image stitching algorithm is particularly dependent on the number and quality of feature points, so it has a good effect on image stitching with more feature points. However, this algorithm’s effect on images with fewer feature points is poor [19]. To date, there is still no mature theory or technology for small-scale image stitching, but the method based on external feature points offers the possibility to register images lacking their own feature points [20]. The surface root image features of grafted seedlings are relatively monotonous, and the two-dimensional code pattern is used as the background to provide sufficient feature points. As shown in Figure 2, Harris corner detection [21], feature descriptor extraction, and feature matching [22] are used to realize the stitching of surface root color images. After that, use of the adaptive non-maximal suppression (ANMS) [23] method and outlier removal based on random sample consensus (RANSAC) can help obtain a more accurate homography matrix. Finally, based on this matrix, the four-view color images can be transformed into a single intact image to complete the mosaic of color images of surface roots. For stitching of the depth map, the official alignment function provided by Microsoft is used here to align the depth map to the color map, and then the homography matrix calculated above is used to complete the stitching of the depth maps. The region of interest (ROI) is set in the RGBD image, and the sub-images containing the surface roots are output for subsequent processing.

### 2.4. Precision Segmentation Method for the Surface Root Image of Root Ball of Plug Seedling

#### 2.4.1. Surface Root Image Preprocessing for Root Ball of Plug Seedling

Step 1: Image enhancement. First, we increase the light intensity of the stitched surface root color image and then use Gamma transform to improve the contrast of the image.

Step 2: Background removal based on edge. The Sobel operator is then used to extract the edges, and the disk structure element is used to extend the pixels (dilate the image) to the extracted edges. Then, we can use hole filling based on morphology [24], corrosion based on diamond structure elements, and open operation based on disk structure elements. Next, the pixel area of the connected domain is detected, and the connected domain, less than half of the total pixel, is removed to obtain images that contain only the surface roots or only the soil matrix.

**Figure 3:**
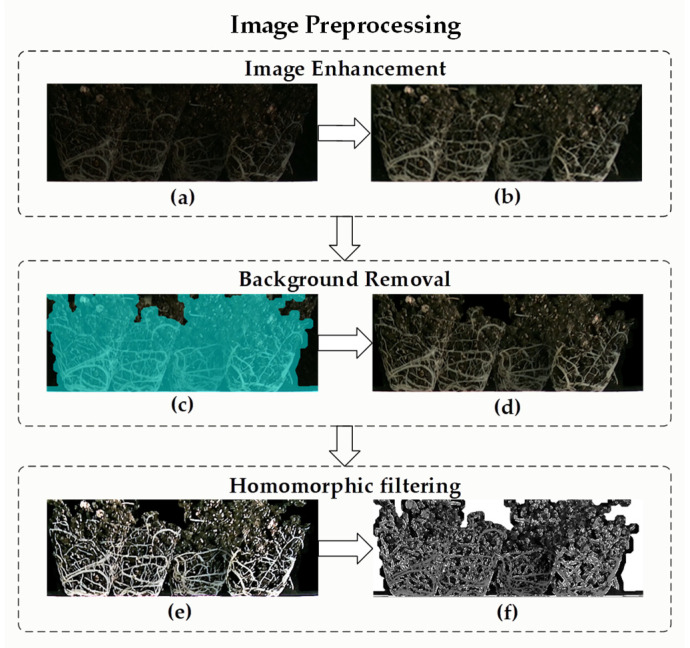
Image preprocessing. (**a**) Completed mosaic of color images. (**b**) Images with enhanced brightness and contrast. (**c**) High-brightness display of foreground and background segmentation results. (**d**) Removed background image. (**e**) Morphologically enhanced images. (**f**) The resulting image after homomorphic filtering.

Step 3: Homomorphic filtering. To reduce the influence of light, as shown in Figure 3, we can attenuate the low-frequency components through homomorphic filtering, enhance the high-frequency parts of the image, and effectively retain fine roots.

#### 2.4.2. Surface Root Image Segmentation Algorithm

**Figure 4:**
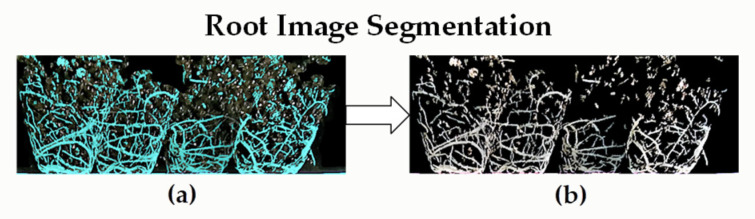
Root image segmentation. (**a**) High-brightness comparison between surface root and substrate. (**b**) Images of surface root extraction results.

As shown in Figure 4, the root system of plug seedlings has a local linear structure, and the root thickness is different, so the Hessian matrix is sensitive to the linear segment structure for detecting the root system. That is, we can use the Gaussian functions of different scales (G(x,y;σ)) to smooth the preprocessed rootstock root image and then calculate the filtered Hessian matrix. Then, eigenvalues $\lambda_{1}$ and $\lambda_{2}$($\lambda_{1}$<$\lambda_{2}$) of the Hessian matrix at each scale can be calculated. With the Gaussian smoothing parameter (*σ*) as the standard deviation, for the linear structure of roots, when the scale factor most strongly matches the actual width of roots, the output of the filter is the largest. Thus, as a spatial scale factor, iteration can obtain outputs at different scales. The half width of the window rectangle for local characteristic analysis is generally 3σ. When the root diameter is smaller than the width and height of the corresponding window rectangle at the current scale, the eigenvalues of the Hessian matrix of tubular roots satisfy the following formula:

(1) $$\lambda_{1}\approx 0,\lambda_{1}\leq \lambda_{2}$$

Moreover, the Hessian matrix is a symmetric matrix. Through feature decomposition of the Hessian matrix, two eigenvalues and corresponding eigenvectors can be obtained. The eigenvectors of the Hessian matrix satisfy the orthogonal relationship, and the two eigenvalues can represent the intensity of the root image. The distribution of the root is tubular, and the change in gray level along the root direction is small; thus, the second-order differential response in the Hessian matrix is also small. The second-order differential response of the Hessian matrix is stronger when the gray level changes greatly across the root direction. The matrix background is a relatively uniform smooth region, and the second-order differential response in the Hessian matrix is also small. That is to say, the eigenvalues of the Hessian matrix are large or small at the root pixels. At the root intersection, the two eigenvalues of the Hessian matrix are larger; at the background point, the eigenvalues of the Hessian matrix are small.

According to the filtering method proposed by FRANGI, we obtained the root pixel response function and then obtained the root pixel to propose the matrix pixel. As shown in Equations (2) and (3), $\beta \in \left[0.3,2\right],\;c\in \left[10^{-5},\;10^{-6}\right]$ can be used to adjust the difference between bulk impurities such as linear rootstock roots and matrix foam rocks. Here, *c* is the parameter that controls the overall smoothness of linear objects. The half width of the window rectangle of pixel p in the root map with the matrix is generally 3σ. When the window corresponding to different diameters matches the root diameter, v(S) will obtain the maximum response v(p):

(2) $$R_{B}=\frac{\left|\lambda_{1}\right|}{\left|\lambda_{2}\right|},S={\left\|H\right\|}_{F}=\sqrt{\sum_{j\leq D}\lambda_{j}^{2}}$$

(3) $$V\left(S\right)=\{\begin{array}{c} 0,\;\;if\;\lambda_{2}>0,\\ \exp\left(-\frac{R_{B}^{2}}{2\beta^{2}}\right)\left(1-\exp\left(-\frac{S^{2}}{2c^{2}}\right)\right) \end{array}$$

where $R_{B}$ is the Linearity measure in 2D and accounts for the eccentricity of the second order ellipse, and S represents the norm of all eigenvalues.

The response function of the root pixels belonging to pumpkin rootstock in multi-scale is as follows:

(4) $$v\left(p\right)=\underset{\sigma \in \left[\sigma_{\min},\sigma_{\max}\right]}{\max}V\left(S\right)$$

### 2.5. Phenotypic Data Mining, Non-Destructive Measurement, and Accurate Estimation

Key phenotypes of rootstock root phenotypes include the average diameter of total root (ADTR, μm), surface area of surface root (SASR, cm^2^), density of surface root (DSR), length of total root (LTR, cm), and volume of total root (VTR, cm^3^). Among them, the average diameter of total root, surface area of surface root, and density of surface root could be directly calculated using the algorithm in this paper, while prediction models were established for other phenotypes to provide estimations.

#### 2.5.1. Non-Destructive Measuring Method for Average Diameter of Total Root

**Figure 5:**
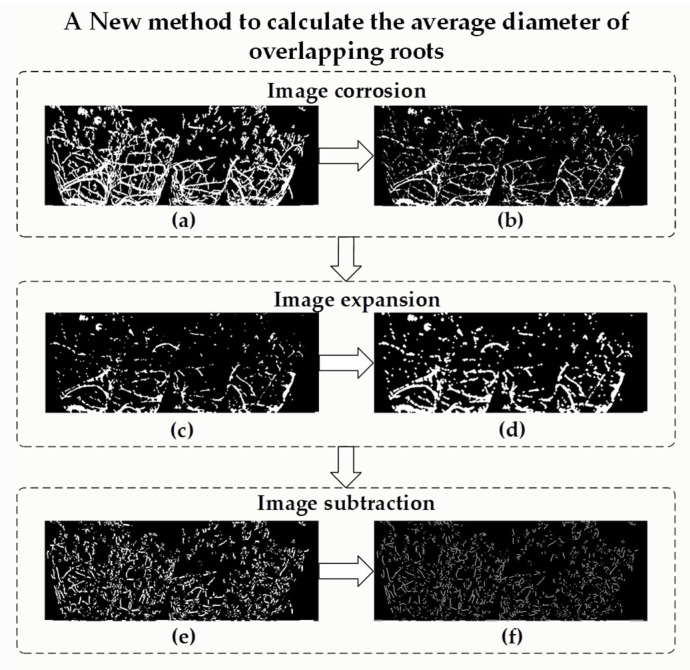
A new method to calculate the average diameter of overlapping roots: (**a**) Binary root image. (**b**) Binary image after corrosion. (**c**) Median filtering. (**d**) Expanded image. (**e**) Binary image minus expansion image. (**f**) Skeleton extraction.

The average diameter of total root is a key phenotypic parameter that represents the potential ability of roots to penetrate the soil and branches through hydraulic conductivity. Root diameter is also the focus of this measurement. There is surface root overlap in plug seedlings, and the stronger the root is, the more serious the overlap will be. There are many overlapping and parallel roots in the surface layers of root nodules, which makes it difficult to estimate the average diameter. In light of this issue, we proposed an estimation method for overlapping roots, as shown in Figure 5. Here, there is an important feature in the surface root image. The non-overlapping roots (hereafter referred to as non-overlapping roots) in the surface root image cannot be reproduced by expansion after corrosion, and the overlapping roots can be restored to the original pixel size after expansion. Based on this feature, firstly, the non-overlapping roots were removed using a morphological operation, and the non-overlapping root images in the surface root image were retained. Then, the surface root image and the overlapping root image were subtracted to obtain an approximate image of non-overlapping roots.

Firstly, the internal parameters of the depth camera were obtained via calibration, and then the RGBD image of the root *ROI* was transformed into a point cloud. The actual area of the *ROI* ($S_{ROI}$) was calculated according to the point cloud, and then the unit area of the pixel and the unit distance between the pixels were calculated according to Equations (5) and (6):

(5) $$S_{Pix}=\frac{S_{ROI}}{\sum Pix_{}}$$

(6) $$L_{pix}=\sqrt{S_{Pix}}$$

where $S_{ROI}$ is the actual area of the *ROI*, ∑Pix is the total number of pixels in the *ROI*, $S_{Pix}$ is the actual area of a single pixel, and $L_{pix}$ is the length of unit pixels.

In the second step, the skeleton of non-overlapping roots was extracted to obtain the pixel length of the skeleton as the length of non-overlapping roots, as shown in Equation (7):

(7) $$TLSR_{NO-\mathrm{skeleton}}=\sum Pix_{NO-\mathrm{skeleton}}\times 1.2L_{pix}$$

where $TLSR_{NO-skeleton}$ is the total length of the surface non-overlapping root skeleton, and $\sum Pix_{NO-skeleton}$ is the total number of pixels of the non-overlapping root skeleton. Because the skeleton length is calculated here according to the pixel diagonal and the pixel edge length, the average pixel edge length and pixel diagonal length is taken (1.2 times $L_{pix}$).

In the third step, the diameter of non-overlapping roots can be defined as the ratio of non-overlapping root area to non-overlapping root length, which can be used as the average diameter of roots, as shown in Equations (8) and (9):

(8) $$SASR_{NO}=S_{Pix}\times \sum Pix_{NO}$$

(9) $$ADTR\approx ADSR_{NO}=\frac{SASR_{NO}}{TLSR_{NO}}=\frac{SASR_{NO}}{TLSR_{NO-\mathrm{skeleton}}}$$

where $SASR_{NO}$ is the surface area of surface non-overlapping root, ADTR is the average diameter of total root, $ADSR_{NO}$ is the average diameter of surface non-overlapping root, and $TLSR_{NO}$ is the total length of surface non-overlapping root.

#### 2.5.2. Calculation of Root Encapsulation (Density of Surface Root)

Root encapsulation (density of surface root) refers to the ability of plant roots to encapsulate the soil matrix and also represents the size of root–soil contact. As the main determinant for the whole root’s ability to absorb water and nutrients, this parameter can be defined as the ratio of the surface area of surface root to the surface area of the soil matrix, as shown in Equation (10):

(10) $$DSR=\frac{SASR}{SSAM}\times 100\%=\frac{\sum Pix_{r}}{\sum Pix_{a}}\times 100\%$$

Equation (10) is the calculation equation for root encapsulation, namely, the density of surface root, where DSR is the density of surface root, SASR is the surface area of surface root, and SSAM is the surface area of the surface matrix; $\sum Pix_{r}$ is the sum of the surface root image pixels extracted from the matrix; and $\sum Pix_{a}$ is the sum of the root pixels with the surface substrate.

#### 2.5.3. Prediction Model for the Surface Area and Length of Total Root

1. Estimating the Surface Area of Total Root (*SATR*)

**Figure 6:**
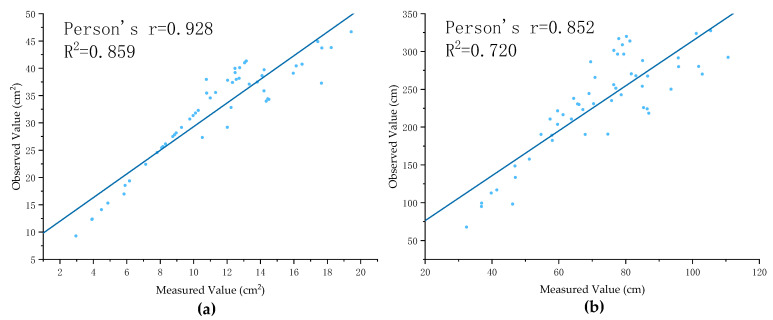
(**a**) Prediction model for the surface area of total root. (**b**) Prediction model for the length of total root.

Using the density of surface root (DSR) obtained above, the surface area of the surface roots can be obtained according to Equation (11):

(11) $$SASR=DSR\times S_{a}$$

where SASR is the surface root surface area, and $S_{a}$ is the surface soil area of plug seedlings. We used the linear regression method to establish the prediction model for the non-destructive measured value/observed value of the surface area of total root, and the fitting results are shown in Figure 6a. The equation for estimating the surface area of total roots is as follows:

(12) SATR=2.16556×SASR+7.6522

2. Estimating the Length of Total Root (*LTR*)

The calculation equation for the length of total root (LTR) is provided in Equation (13):

(13) $$LTR=\frac{SATR_{P}}{ADTR}$$

where $SATR_{P}$ is the predicted value for the surface area of total root, and ADTR is the average diameter of total root.

We used the linear regression method to establish a prediction model for the non-destructive measured value/calculated value of the complete root length, and the fitting results are shown in Figure 6b.

The length of total root (LTR) estimation equation is as follows:

(14) $$LTR_{P}=2.9723\times LTR+16.83062$$

3. Calculating Volume of Total Root (*VTR*)

The volume of total root (VTR) is calculated using Equation (15):

(15) $$VTR=\pi {\left(\frac{ADTR}{2}\right)}^{2}\times LTR_{P}$$

where VTR is the volume of total root, ADTR is the average diameter of total root, an $LTR_{P}$ is the length of total root.

## 3. Results

### 3.1. Performance Test of Images Using the Surface Root Segmentation Algorithms

**Figure 7:**
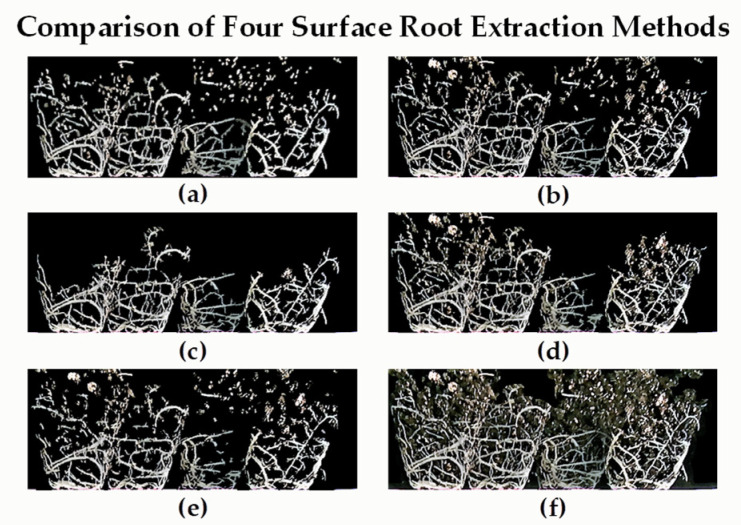
Comparison of surface root extraction methods. (**a**) Artificially extracted root images of Qingyan Rootstock NO.1. (**b**) The proposed algorithm. (**c**) Gabor-filtering-based FRANGI segmented images. (**d**) K-means unsupervised clustering method segmented images. (**e**) Genetic algorithm based on maximum interclass variance method segmented images. (**f**) Unsegmented images.

To test the performance of image segmentation, we used four common image segmentation algorithms to compare the results for artificial root extraction, including the proposed algorithm, Gabor-filter-based FRANGI extraction method, K-means unsupervised clustering method, and genetic-algorithm-based maximum inter-class variance method. The experimental results for Qingyan Rootstock NO.1 are shown in Figure 7. Four groups of RGBD images were taken by manually rotating each plant sample 90° and then analyzing the results. The results show that the proposed method can better remove the matrix while retaining fine roots, and produces an optimal segmentation effect.

### 3.2. Measurement Results and Analysis of Average Diameter of Pumpkin Rootstock

**Figure 8:**
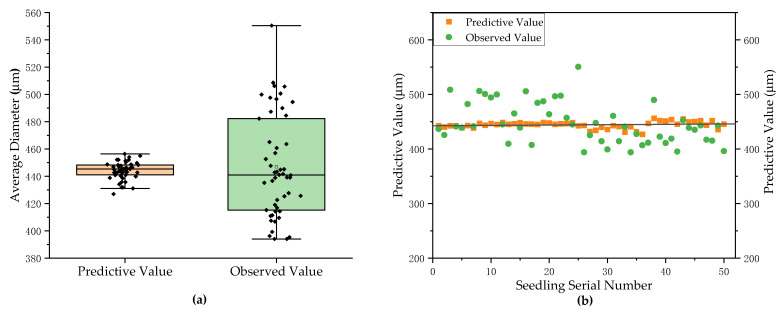
The average diameter of total root measurement results and analysis chart. (**a**) Comparison of the mean values of measured predictive and observed root diameters. (**b**) Deviation of the measured average diameter of total root.

Next, we expanded the analysis to six pumpkin rootstock varieties. As shown in Figure 8, analysis of 50 randomly selected groups among the six pumpkin rootstock varieties yielded an average diameter measurement accuracy of 93.3%, a maximum measurement error of 65 μm, a minimum measurement error of 2 μm, and an average measurement error of 29 μm. The average diameter of the total root measurement method proposed in this paper offers good measurement accuracy and can completely replace the use of a root scanner for non-destructive measurements.

### 3.3. Prediction Results and Analysis of Total Root Phenotypes of Pumpkin Rootstocks

**Figure 9:**
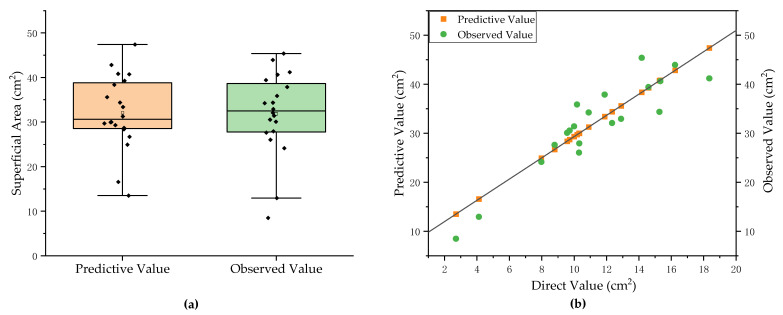
Prediction results and analysis of surface area of total root. (**a**) Comparison between the predictive and observed value. (**b**) The deviation of predictive value of surface area of total root.

The prediction model based on the average root diameter of pumpkin rootstock can estimate five key agronomic parameters, including surface area, diameter, volume, and encapsulation. As shown in Figure 9, the measured values for the surface area of total root among the 20 groups of pumpkin rootstocks were randomly selected and input into the prediction model. The prediction accuracy of the predicted value reached 88.1%, the maximum measurement error was 7.022 cm^2^, the minimum prediction error was 0.159 cm^2^, and the average prediction error was 3.083 cm^2^. The experimental results showed that the surface area of surface root is highly correlated with the surface area of total root and that the surface area estimation method of total root proposed in this paper offers good accuracy.

**Figure 10:**
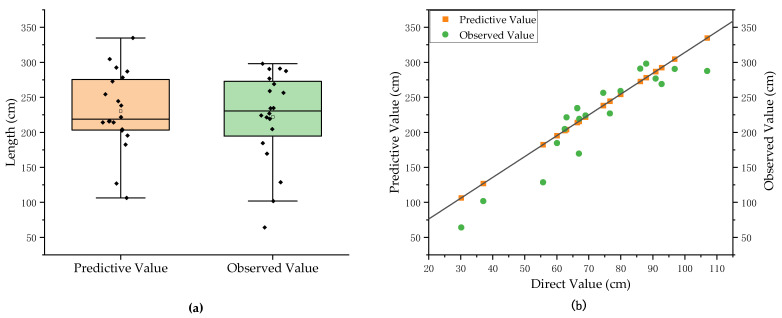
Prediction results and analysis of length of total root. (**a**) Comparison of predictive and observed value. (**b**) The deviation of predictive value of length of total root.

The measured values for length of total root among the 20 groups of pumpkin rootstocks were randomly selected and input into the prediction model. As shown in Figure 10, the prediction accuracy of the predicted value reached 87.2%, with a maximum prediction error of 36.06 cm, a minimum prediction error of 2.24 cm, and an average prediction error of 20.82 cm. Considering the inevitability of such errors, we believe that the non-destructive measurement of the length of total root of pumpkin rootstocks basically meets the measurement requirements.

## 4. Discussion

Due to the characteristics of stress resistance and vigorous growth, pumpkin is often used as the rootstock of watermelon and cucumber and is beneficial for the absorption of nutrients and water in grafted watermelon and other crops. The root phenotype of the rootstock is closely related to the water absorption and resistance of grafted seedlings. Root phenotype parameters can be used to evaluate root morphology and judge the quality of rootstocks. Root scanners, which are destructive and tedious to use, are currently widely employed to measure pumpkin rootstock phenotypes. In this study, we proposed a non-destructive, easy-to-implement, high-throughput method for measuring the root phenotypes of pumpkin rootstocks. This method is expected to advance root-related research. In addition to directly measuring the average diameter of the superficial root system, this method also estimates the surface area and length of the complete root system, and the overall measurement accuracy is very good. Several key considerations in this study are discussed below.

### 4.1. Discussion of the Root Image Stitching Algorithm

The effect of image stitching is very important and directly affects the measurement accuracy of the root phenotype. The image mosaic method based on the background of the two-dimensional code pattern used in this paper is simple and effective. However, inevitably, the axis of the root system was not oriented in a straight line during the rotation process, which led to the four images of the root system having different degrees of difference relative to the two-dimensional code pattern. This factor led to certain errors in the image stitching results, especially the stitching of the first and fourth images, which presented a small amount of residual overlapping areas. The use of electromechanical devices for stable rotation of the root system can reduce such errors. It is necessary to further develop an automated root mapping device and supporting software system to prepare for large-scale root phenotype measurements.

### 4.2. Discussion of the Root Image Segmentation Algorithm

The vermiculite, perlite, and other substances in the matrix can be similar in color to the root system, leading to parts of these impurities being mistakenly divided into the root system, resulting in the measured value of the root phenotypic parameters being too large. Using colored perlite or similar materials can eliminate this error. Conversely, we could explore the use of more advanced algorithms, such as deep learning methods, for more precise segmentation.

### 4.3. Discussion on the Calculation Method for the Root Phenotype

Although the non-overlapping part of the superficial root system has a certain representativeness, there is also a certain degree of chance, as well as unavoidable slight errors in estimating the average diameter of the root system using this part. Other root phenotypes are based on data for the average diameter and, therefore, have a range of errors. However, based on the experimental results, this method still offers high measurement accuracy. In addition, a linear fitting method was used in this study to estimate the parameters of the complete root system. Whether there are different fitting methods for the root system in different growth stages needs to be further explored.

### 4.4. Discussion of Method Adaptability

Seedling methods, seedling varieties, cultivation time, and other factors will lead to changes in the root systems of seedlings. The six cultivars used in this experiment had good measurement accuracy, which demonstrated the effectiveness of the method outlined in this paper. Whether the non-destructive testing method for pumpkin seedling roots can be directly applied to other seedlings remains to be verified by future experiments.

## 5. Conclusions

The method proposed in this paper can measure seedling root phenotypes in a high-throughput and non-destructive manner, and the measurement accuracy was comparable to that of a root scanner, which can meet the needs of seedling root research. By using a depth sensor to acquire multiple color and depth images of root stocks and then removing the overlapping areas of the multi-view images through stitching and fusion, the superficial roots in the images were segmented, and a series of phenotypic parameters were measured. Among these parameters, the encapsulation and average diameter of total root were the results of direct calculations. The measurement accuracy of the most critical average diameter of total root reached 93.3% with deviation of ±0.03 mm (30 μm). Using these measured data and the artificially measured phenotype data of the intact root for fitting, the residuals of the fitting were all less than 10%, which demonstrates the strong correlation of the surface area and predictability of other measurement parameters between the surface root system and total root system. This study provides a new direction for non-destructive measurement of the pumpkin rootstock root phenotype and will provide key basic data for root growth research of rootstock.

## Data Availability

The data presented in this study are available on request from the corresponding author.
